# Site-specific scFv labelling with invertase via Sortase A mechanism as a platform for antibody-antigen detection using the personal glucose meter

**DOI:** 10.1038/srep19338

**Published:** 2016-01-19

**Authors:** Nur Faezee Ismail, Theam Soon Lim

**Affiliations:** 1 Institute for Research in Molecular Medicine, Universiti Sains Malaysia, 11800 Penang, Malaysia

## Abstract

Antibody labelling to reporter molecules is gaining popularity due to its many potential applications for diagnostics and therapeutics. However, non-directional bioconjugation methods which are commonly used often results in the loss of target binding capabilities. Therefore, a site-specific enzymatic based bioconjugation such as sortase-mediated transpeptidation allows for a more rapid and efficient method of antibody conjugation for diagnostic applications. Here we describe the utilization of sortase A bioconjugation to conjugate a single chain fragment variable (scFv) to the extracellular invertase (invB) from *Zymomonas mobilis* with the aim of developing an invertase based immunoassay. In addition, conjugation to enhanced green fluorescent protein (eGFP) was also validated to show the flexibility of the method. The invertase conjugated complex was successfully applied for the detection of antibody-antigen interaction using a personal glucose meter (PGM) for assay readout. The setup was used in both a direct and competitive assay highlighting the robustness of the conjugate for assay development. The method provides an alternative conjugation process to allow easy exchange of antibodies to facilitate rapid development of diagnostic assays for various diseases on the PGM platform.

An array of sensing technologies have been developed allowing users the freedom to detect target molecules either by various methods including colorimetric, fluorescence, electrochemistry and label free methods^1^,^2^,^3^. However, a common complication with these methods is the need for laboratory-based instrumentations or even customized devices to be used. Traditionally, antibody-antigen detection systems are designed mainly using colorimetric or fluorescent based readouts^1^. Such methods require either the antibody or antigen to be chemically labelled with dyes or biological fusion constructs such as fluorescent proteins or even enzymes like alkaline phosphatase^4^,^5^,^6^. Conventional conjugation methods utilizing reactive functional groups such as NHS-ester maleimide-mediated conjugation with heterobifunctional cross linker containing both amine-reactive NHS ester and sulfydryl maleimide^7^, glutaraldehyde-mediated conjugation with a stable secondary amine linkage^8^ and reductive amination-mediated conjugation^9^, and newer methods such as click chemistry^10^,^11^,^12^ are commonly used. The major setback to these conventional chemical bioconjugation processes is the potential loss of biological function of the protein as chemical attachment of the reporter is random. Therefore, a biologically friendly conjugation method with site-specificity is desirable for protein-protein attachments.

Sortase A functions *in vivo* to attach proteins covalently to the bacterial cell wall. During sortase A transpeptidation, the Cys^184^ with His^120^ and Arg^197^ in proximity within the hydrophobic region of the β6/β7 loop of the Sortase A active site is utilized to interact with the LPXTG motif protein^13^,^14^. This LPXTG motif is then cleaved at the carbonyl group between threonine and glycine forming an intermediate thioacyl complex. The complex is then resolved by a nucleophilic attack of the activated N-terminal oligoglycine protein thus releasing the fusion protein. Naturally, Sortase A is directly related to the pathogenicity of Gram positive bacteria by sorting and attaching the virulent factor to the lipid II of bacteria. These virulent factors known as microbial surface component recognizing adhesive matrix molecules are important in adherence of the bacteria to host cell and infection. The carboxyl terminus of the cleaved product would chemically link with the terminal amino group of a penta-glycine linker in the peptidoglycan. This natural adaptation has been used successfully *in vitro* to link various compounds that exhibit the C-terminal LPXTG motif under mild conditions^15^. This strategy has been well adapted for use in fluorescent labelling for sensing applications^16^,^17^,^18^,^19^.

The personal glucose meter (PGM) has been a revelation in the health care system allowing simple point-of-care (POC) monitoring of glucose levels for diabetics. The PGM is an attractive tool for POC applications due to its compact size, low cost, reliability and simple operation procedures. The evolution of the PGM as a biosensor is evident with reports showing the application of PGM for the detection of small molecules, proteins, pathogens, metal ions and even nucleic acid^20^,^21^,^22^,^23^,^24^. The basis of the detection is centred on the presence of a sucrose hydrolysing enzyme, the extracellular invertase, invB from *Zymomonas mobilis*^25^,^26^. The hydrolysis of sucrose by invB results in the production of glucose and the conversion levels can be monitored using a PGM. Most studies incorporating invB as a reporter molecule applied chemical-based conjugation methods or intermediary anchor molecules such as the biotin-avidin interaction to chemically attach invB to their target molecules^20^,^21^,^22^. The application of sortase A based conjugation of single chain fragment variable (scFv) with invB has not been reported before.

Here we propose the application of Sortase A transpeptidation to conjugate invB to recombinant scFv antibodies for application on the PGM as a biosensor to detect antibody-antigen interactions. Figure 1 shows the overall process of Sortase-mediated conjugation of scFv to invB and the application of the conjugated product to detect antigens using a commercial PGM. The concept allows the use of Sortase A to conjugate recombinant antibodies with invertase to function as a tagged antibody using the PGM. Target antigens will be coated on the surface of microtitre plates to allow capture by invertase tagged antibodies. A wash step will remove all unbound antibodies leaving the captured tagged antibodies for sucrose conversion. The conversion of sucrose to glucose would then lead to readouts detectable using the PGM. This permits the application of sortase mediated conjugation for simple and efficient antibody labelling virtually against any reporter protein. The adaptation of the method for invB to be applied on PGM allows the development of a cheap and effective alternative POC assay for use in resource limited settings.

## Result and Discussion

In this study, anti-ubiquitin scFv (Ubi scFv) was used as the model protein. In order to investigate the flexibility of the system, two different systems were prepared. The model Ubi scFv (29.97 kDa) derived from panning of an in-house human naïve scFv phage display library was conjugated to invertase (45.8 kDa) and eGFP (26.4 kDa). The Ubi scFv vector was modified to include the LPETGG and avidin tag as fusion to the scFv. The avidin tag was included to facilitate a two-step purification process downstream post-hexahistidine purification. Meanwhile, eGFP and invB were tagged with both oligoglycine (G_x_) and hexahistidine (H_6X_) tag.

### Determination of protein concentration

**Figure 2:**
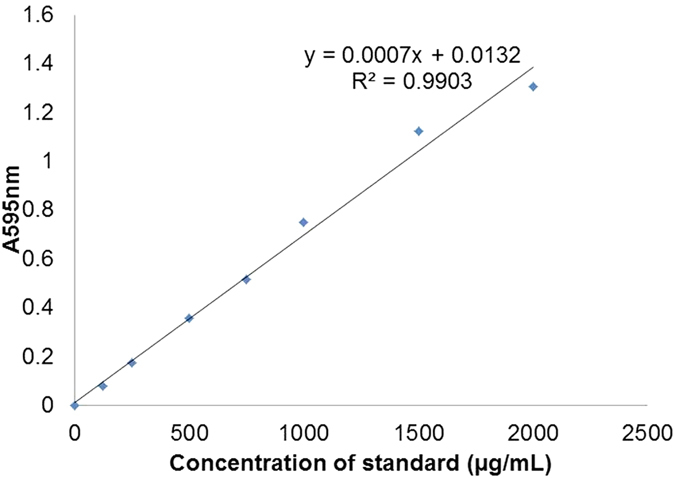
Standard curve plotted to determine the concentration of SrtA conjugation components.

To determine the concentration of the proteins, Quick Start™ Bradford Protein Assay (Bio-Rad) was used. Figure 2 shows the standard curve plotted to determine the concentration of SrtA conjugation components. In this study, only standard curves with R^2^ value of 0.98 to 0.99 was used. The expressed and purified fractions of 100 mL of invertase, eGFP, Ubi scFv and sortase A was determined to be ~3.03 mg/mL for invertase, ~3.9 mg/mL for eGFP, 1.0 mg/mL for Ubi scFv and 9.4 mg/mL for SrtA.

### Conjugation of Ubi scFv and eGFP

**Figure 3:**
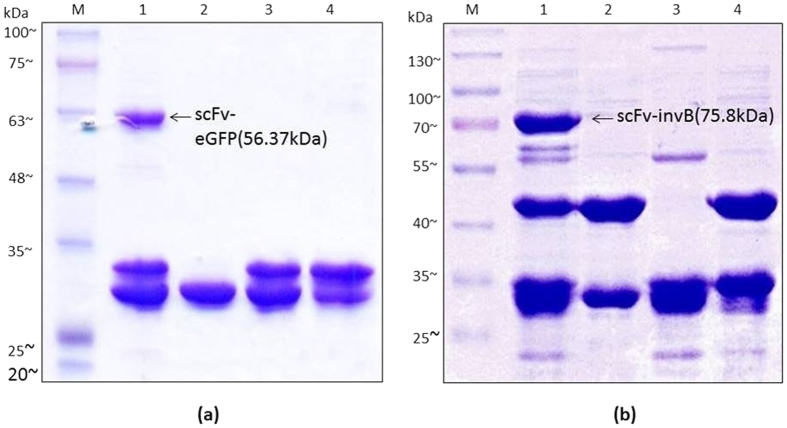
SDS-PAGE analysis of conjugation at optimal condition. (**a**) SDS-PAGE analysis of Ubi scFv-LPETG_5_-eGFP. M: BluEye Prestained Protein Ladder, 1: Conjugation of 5 μM Ubi scFv-LPETGG and 5 μM G_5_-eGFP aided by 5 μM SrtA, 2: Reaction control (5 μM Ubi scFv-LPETGG + 5 μM G_5_-eGFP), 3: Reaction control: (5 μM G_5_-eGFP + 5 μM SrtA), 4: Reaction control: (5 μM Ubi scFv-LPETGG + 5 μM SrtA) (**b**) SDS-PAGE analysis of Ubi scFv-LPETG_5_-invB. M: BluElf Prestained Protein Ladder, 1: Conjugation of 5 μM Ubi scFv-LPETGG and 5 μM G_5_-invB aided by 5 μM SrtA, 2: Reaction control (5 μM Ubi scFv-LPETGG + 5 μM G_5_-invB), 3: Reaction control: (5 μM Ubi scFv-LPETGG + 5 μM SrtA), 4: Reaction control: (5 μM G_5_-invB + 5 μM SrtA).

Conjugation of Ubi scFv to to eGFP was carried out as a positive control for Sortase A conjugation. The purpose of this test was to ensure the functionality of the Sortase A mediated conjugation using the extracted *srt*A gene product from *Staphylococcus areus* ATCC 25923. The optimization steps used were based on previously published methods^15^,^19^,^27^,^28^. To achieve an optimal conjugation condition, factors such as motif efficiency, temperature, reaction pH, CaCl_2_ concentration, incubation time and ratio of reactant to enzyme were tested. The results of the optimization are shown in Supplementary Data. For the conjugation of Ubi scFv and eGFP, the optimized condition was established using G_5_-eGFP and Ubi scFv-LPETGG, at 37˚C, with 3 h incubation time using 1 to 1 ratio of reactants to enzyme, 5 mM CaCl_2_ and buffer condition at pH 7.5. Figure 3(a) shows the SDS-PAGE analysis of the conjugation reaction of Ubi scFv-LPETGG and G_5_-eGFP at the optimized condition. The conjugation of Ubi scFv-LPETGG and G_5_-eGFP served as a control reaction for the Sortase A conjugation mechanism. Based on the analysis, in the optimized reaction condition, the conjugated product with an estimated size of ~56.4 kDa was only present at the reaction lane containing 5 μM of Ubi scFv-LPETGG, 5 μM of G_5_-eGFP and 5 μM of SrtA.

### Conjugation of Ubi scFv and invB

Since the Sortase A mechanism is protein-dependent^19^, optimization of the conjugation conditions for Ubi scFv and invB was conducted. The best condition for Ubi scFv and G_5_-invB conjugation was established at 25 °C, pH 6.0 with 5 mM CaCl_2_ using a 1 to 1 ratio of enzyme to reactant in 3 h incubation. Figure 3(b) shows the SDS-PAGE analysis of the conjugation between scFv and invB at its optimal condition. Lane 1 represents the reaction lane where the calculated size of unconjugated Ubi scFv-LPETGG size was ~29.97 kDa and the calculated size of unconjugated G_5_-invB was ~45.8 kDa. The estimated size of Ubi scFv-LPETG_5_-invB is at 75.8 kDa. Based on the results, with respect to the controls, only reaction at Lane 1 produced the band at the expected size. The conjugation with invB showed the formation of two weak byproduct bands similar to the Sortase A reaction of Ubi scFv-LPETGG and G_5_-eGFP. For Ubi scFv and eGFP reaction, the byproduct formation spotted was lesser and most of the time is not clear compared to the reaction of Ubi scFv and G_5_-invB. This byproduct formation could be due to the ability of sortase A to initiate hydrolysis.

Hydrolysis of activated acyl donor can occur in aqueous reactions resulting in the formation of unwanted byproducts^29^. The effects of hydrolysis will result in lower yields of conjugated product. Sortase A catalyzed conjugation of antibodies namely the Fab fragment has been reported to result in hydrolysis of the sorting motif and unspecific covalent crosslinking of the light and heavy chains^30^. The formation of the byproduct increased when there is no acceptor nucleophiles present in the reaction^31^ which was seen in this experiment where both reactions without the addition of G_5_-eGFP or G_5_-invB in the reaction formed a byproduct at the size of ~55 kDa suggesting the existence of Ubi scFv-SrtA complex resulting from hydrolysis. Even so, the amount of conjugated product was sufficient for use and loss of yield was not huge.

### Western blot analysis of conjugated Ubi scFv-LPETG_5_-eGFP and conjugated Ubi scFv-LPETG_5_-invB

**Figure 4:**
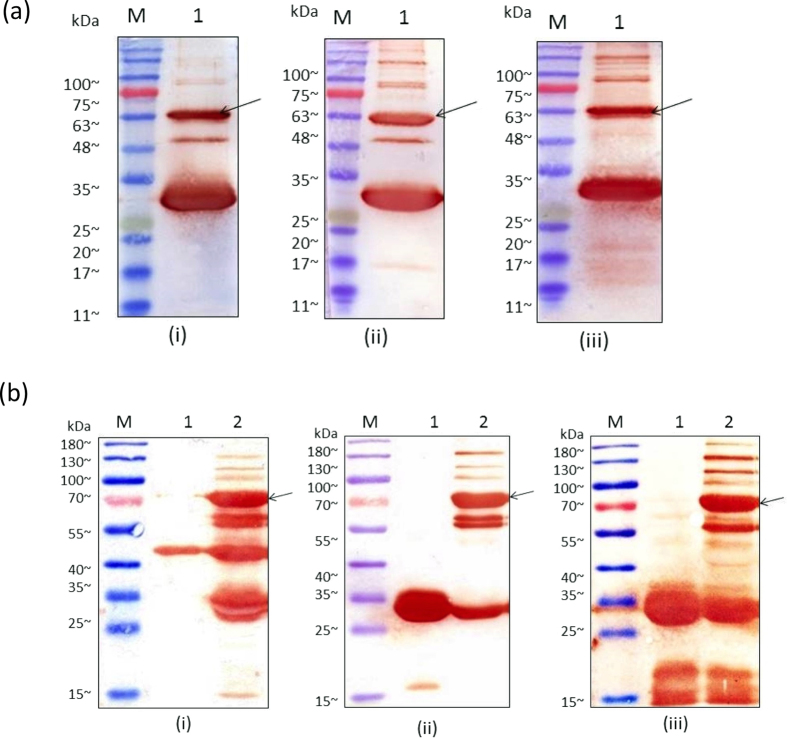
Western blot of conjugated product. (**a**) Ubi scFv-LPETG_5_-eGFP, (**b**) Ubi scFv-LPETG_5_-invB, (i) blotted against protein-L HRP, (ii) blotted against streptavidin HRP, (iii) blotted against anti-His.

To further confirm the formation of the conjugated product, western blot analysis was conducted (Fig. 4(a)). As the reaction contains two different proteins with unique tags fused together, detection was done using several methods. For Ubi scFv-LPETGG, protein-L and streptavidin were used as the tags are only unique to itself in the reaction performed. Protein-L will bind specifically to the scFv containing the kappa-light chain^32^,^33^ whereas streptavidin would only bind to the biotin tag^34^,^35^. The histidine tag found in both eGFP and invB was used for detection using anti-His antibodies. As such, only eGFP, invB and Sortase A will produce a colour development.

**Figure 5:**
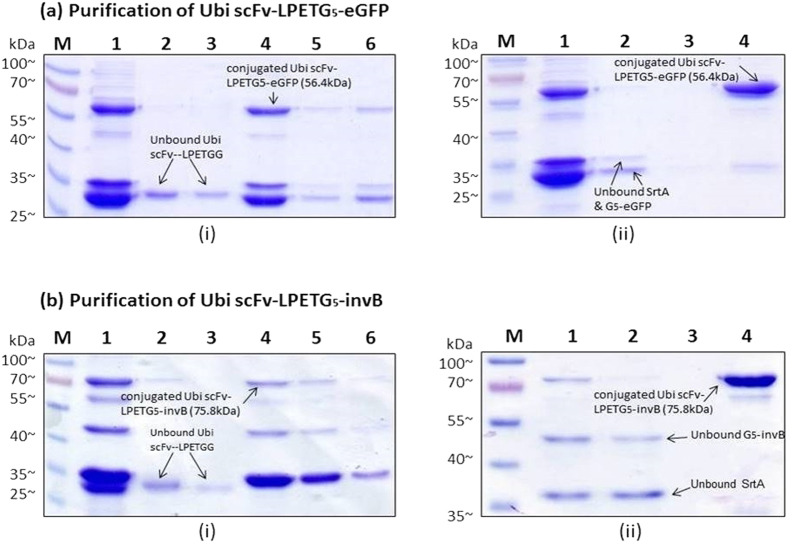
SDS-PAGE analysis of purification process. (**a**) Purification of Ubi scFv-LPETG_5_-eGFP (i) His-tag purification of Ubi scFv-LPETG_5_-eGFP. M: BluElf Prestained Protein Ladder, 1: Conjugation fraction of Ubi scFv-LPETGG and G_5_-eGFP, 2: Flow through fraction of his-tag purification, 3: Wash fraction of his-tag purification, 4,5,6: Eluted purified fraction of Ubi scFv-LPETG_5_-eGFP (ii) Avidin Purification of his-tag purified Ubi scFv-LPETG_5_-eGFP. M: BluElf Prestained Protein Ladder,1: Conjugation fraction of Ubi scFv-LPETGG and G_5_-eGFP, 2: Flow through fraction of Avidin purification, 3: Wash fraction of Avidin purification, 4: Eluted purified fraction of Ubi scFv-LPETG_5_-eGFP. Figure 6(b) Purification of Ubi scFv-LPETG_5_-invB. (i) His-tag purification of Ubi scFv-LPETG_5_-invB. M: BluElf Prestained Protein Ladder, 1: Conjugation fraction of Ubi scFv-LPETGG and G_5_-invB, 2: Flow through fraction of his-tag purification, 3: Wash fraction of his-tag purification, 4,5,6: Eluted purified fraction of Ubi scFv-LPETG_5_-invB. (ii) Avidin Purification of his-tag purified Ubi scFv-LPETG_5_-invB. M: BluElf Prestained Protein Ladder,1: Conjugation fraction of Ubi scFv-LPETGG and G_5_-invB, 2: Flow through fraction of Avidin purification, 3: Wash fraction of Avidin purification, 4: Eluted purified fraction of Ubi scFv-LPETG_5_-invB.

In Ubi scFv and eGFP conjugation, the western blot analysis showed a colour development at ~56.4 kDa for all the reaction using anti-His, protein-L and streptavidin-HRP (Fig. 5(a)). Therefore, the observed band was confirmed to be the conjugated product of Ubi scFv-LPETG_5_-eGFP. Meanwhile, Fig. 4(b) shows the western blot analysis of Ubi scFv-LPETGG and G_5_-invB conjugation mediated by SrtA. In Fig. 4(b)(i), colour development was observed at 75.8 kDa in the reaction lane when tested using anti-His antibodies with respect to the positive control of G_5_-invB. Theoretically, only invB and Sortase A would generate a colour development since only these two proteins were tagged with a histidine-tag. Hence, the observed band formed must contain invB. The presence of Sortase A is eliminated since no band at 75.8 kDa was observed when reacted with either one of the motifs only. To further confirm the presence of Ubi-scFv at 75.8 kDa band, western blot was performed with protein-L HRP (Fig. 4(b)(ii)) and streptavidin-HRP (Fig. 4(b)(iii)). A band at the estimated size of 75.8 kDa was also present in both reactions with respect to the Ubi scFv-LPETGG as the positive control. Therefore, we can conclude that the observed 75.8 kDa band is the conjugated product of Ubi scFv-invB.

### Two-step purification of conjugated product

The purpose of introducing different tags to different motifs, for example introducing avidin tag to LPXTG motif protein and hexahistidine tag to the pentaglycine was to facilitate the protein purification process of the conjugated product without any interference from the unreacted protein components. As the reaction of Sortase A can never be absolute^36^,^37^, this two-step purification allows the removal of non-specific and unreacted proteins. The proteins were designed with the avi-tag at the C-termini of Ubi scFv-LPETGG. Meanwhile, the hexahistidine tag was fused at the N-terminal pentaglycine motif in eGFP and invB constructs. The two-step purification requires the his-tag purification to be performed first to remove the unreacted Ubi scFv-LPETGG as it will compete for the active site in the avidin column. This is advantageous as the capacity for the avidin column is smaller at ~1.2 mg/mL compared to the Ni-NTA column for his-tag purification which has a capacity of ~40 mg/mL. This would allow elimination of unreacted Ubi scFv-LPETGG from the reaction efficiently.

For the second step of the purification using the avidin column, the purified fraction from the first purification was used. Figure 5(a)(i) shows the His-tag purification of conjugated product of Ubi scFv-LPETG_5_-eGFP, meanwhile Fig. 5(a)(ii) shows the Avidin purification of Ubi scFv-LPETG_5_-eGFP. In 5(a)(i), the elimination of unreacted Ubi scFv-LPETGG was successful and the second step purification using Avidin purification also eliminated the his-tagged G_5_-eGFP and SrtA. This is due to the specific and strong non-covalent interaction normally associated with biotin and avidin interactions^38^. The collected fractions of purified product was then concentrated using a spin column. A protein standard with R^2^ = 0.9817 was plotted and the concentration of purified Ubi scFv-LPETG_5_-eGFP was quantified to be ~0.6 mg/mL for a 100 μL reaction.

Purified conjugated Ubi scFv-LPETG_5_-invB(75.8 kDa) was also successfully obtained using the two-step protein purification method. Figure 5 (b)(i) shows the His-tag purification of conjugated Ubi scFv-LPETGG_5_-invB, meanwhile Fig. 5(b)(ii) shows the second purification step of the conjugated product using the Avidin column. The purification strategy successfully eliminated the unreacted Ubi scFv-LPETGG, invB and SrtA leaving behind the conjugated product. The strategy was successfully adapted for both eGFP and invB fusion products. Then, a concentration procedure was done to concentrate the purified Ubi scFv-LPETG_5_-invB similar to the purification of Ubi scFv-LPETG_5_-eGFP. The purified Ubi scFv-LPETG_5-_invB was quantified to be ~ 0.41 mg/mL for a 100 μL reaction.

### Western blot of purified conjugated Ubi scFv-LPETG_5_-eGFP and Ubi scFv-LPETG_5_-invB

**Figure 6:**
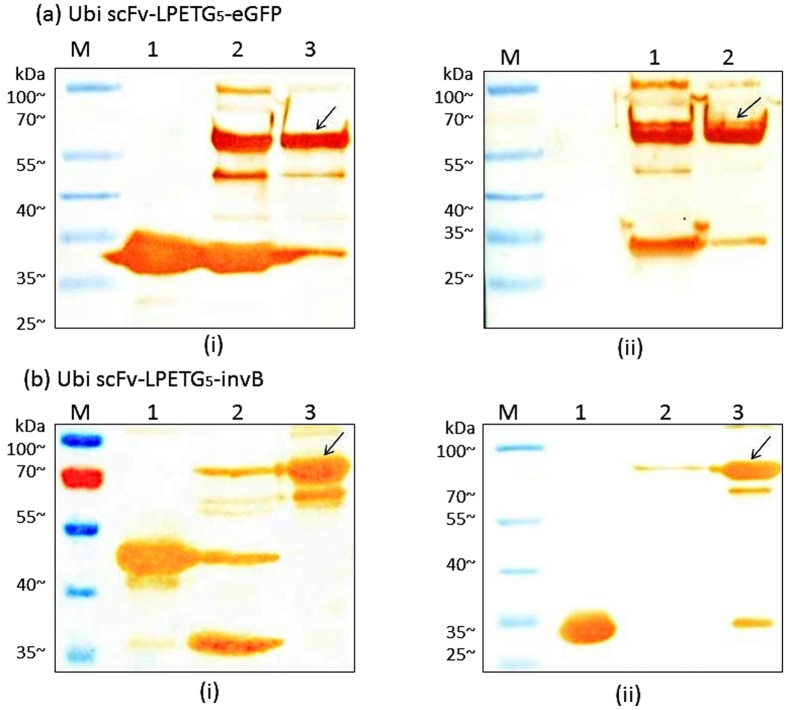
Western blot analysis of purified Ubi scFv-LPETG5-eGFP and Ubi scFv-LPETG_5_-invB. (**a**) Analysis of Ubi scFv-LPETG_5_-eGFP (i) against anti-His, M: BluElf Prestained protein ladder 1: control (eGFP), 2: conjugation reaction of Ubi scFv-LPETGG and G_5_-eGFP, 3: purified conjugated product, (ii) against protein-L M: BluElf Prestained Protein Ladder, 1: conjugation reaction of Ubi scFv-LPETGG and G_5_-eGFP, 2: double-purified conjugated product, (**b**) Analysis of Ubi scFv-LPETG_5_-invB (i) against anti-His M: BluElf Prestained Protein Ladder, 1: control: invB, 2: His-tag purification of Ubi scFv-LPETG_5_-invB, 3: double-purified conjugated product, (ii) against protein-L, 1: control: Ubi scFv, 2: His-tag purification of Ubi scFv-LPETG_5_-invB, 3: double-purified conjugated product.

Western blot analysis was performed to confirm the presence of the conjugated product from the two-step purification strategy. Figure 6 shows the western blot analysis of the purified Ubi scFv-LPETG_5_-eGFP(56.4 kDa) and purified Ubi-scFv-LPETG_5_-invB(75.8 kDa) using anti-His and protein L-HRP as secondary antibodies. The western blot analysis for both samples showed the presence of the conjugated products with respect to the controls. Therefore, the conjugated product was preceded to be tested on the immunoassay platform.

### Limit of detection of Ubi scFv-LPETG_5_-eGFP

**Figure 7:**
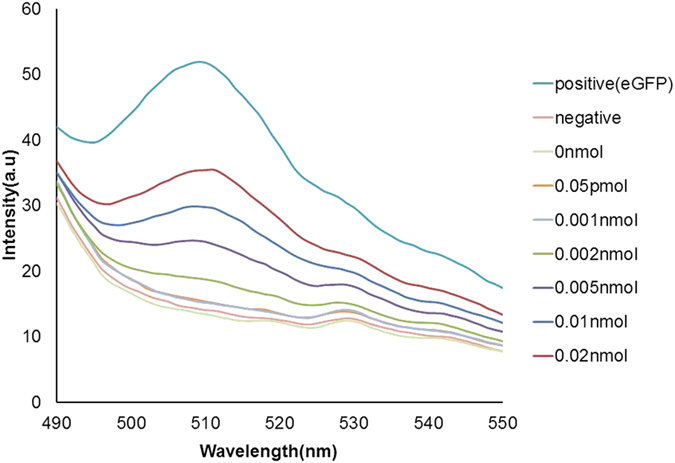
Fluorescent reading of the conjugated Ubi scFv-LPETG_5_-eGFP.

A simple fluorescent immunoassay was carried out to determine the functionality of both the scFv and eGFP conjugate. Various concentrations of ubiquitin (Ubi) at 0 mol, 0.05 pmol, 0.001 nmol, 0.002 nmol, 0.005 nmol, 0.01 nmol, and 0.02 nmol were used for validation. After the addition of samples, the reaction was measured with an excitation wavelength at 460 nm, and emission wavelength set at 400 nm to 600 nm. From the fluorescence spectra analysis, the emission peak of eGFP was detected at 510 nm. Figure 7 shows the fluorescence reading of the conjugated Ubi scFv-LPETG_5_-eGFP. The fluorescent readings showed an increase corresponding with an amount increase of Ubi found on the plate. The readings are proportionate to the amount of Ubi scFv-LPETG_5_-eGFP binding to the antigens. The detection limit of Ubi scFv-LPETG_5_-eGFP was set at 0.05 pmol as it is the lowest detection value achieved. The ability of Ubi scFv-LPETG_5_-eGFP to maintain a fluorescent readout after washing indicates the functionality of both the scFv and eGFP was preserved. This shows the conjugation process using sortase A did not disrupt the folding of the scFv and eGFP. More importantly, the results indicate that no steric hindrance to the binding pocket of the scFv occurred during conjugation.

### Kinetics of sucrose conversion by expressed invertase

**Figure 8:**
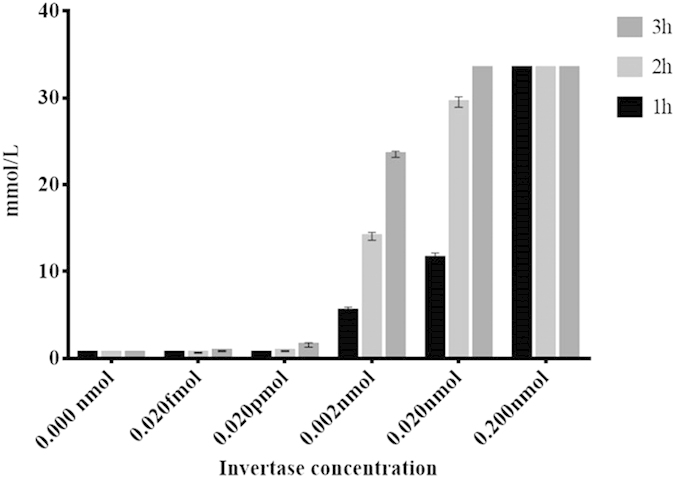
Kinetics of sucrose conversion by expressed Sortase A at an hour (hr) interval. X-axis defines the amount of invB used and Y-axis refers to the glucose reading taken at an hr interval.

The conversion rate of sucrose to glucose by the expressed invertase was monitored using PGM (Accu Chek® Performa) at an hour (hr) interval (Fig. 8). PGM was used for this to determine the minimal amount of enzyme required for a detectable readout. At 0 h, ‘LO’ was shown on the screen that indicates the glucose concentration in the solution is lower than the detection limit of Accu Chek® which is 0.6 mmol/L to 33.3 mmol/L. Meanwhile, ‘HI’ indicates that the glucose concentration in the solution is high and exceeded the maximum detection limit of the meter. In Fig. 8, the reading below 0.6 mmol/L was stated as 0.59 mmol/L as it is the maximum of undetected level of glucose read by the glucose meter and the reading higher than 33.3 mmol/L which exceeded the detection limit was stated as 33.4 mmol/L. Based on the obtained result, 0.020 pmol gave the minimal readout of glucose as early as 1 hr, and 0.020 fmol of invB started to give readings after 2 hr of incubation. Therefore 0.02 pmol was set as the minimum amount of invB to be use for the conversion activity for detection by the PGM in 1 hr. The results suggest the expressed invertase was able to carry out glucose conversion at satisfactory levels which are comparable to that reported in published articles^20^,^21^. The invertase showed a detection limit of 0.02 fmol at two hr incubation with a zero standard deviation of the blank sample. As the pattern of glucose concentration increment per incubation time interval can be observed with respect to the control reaction which indicates a direct correlation between the incubation time to an increase in glucose concentration.

### Limit of detection of Ubi scFv-LPETG_5_-invB

**Figure 9:**
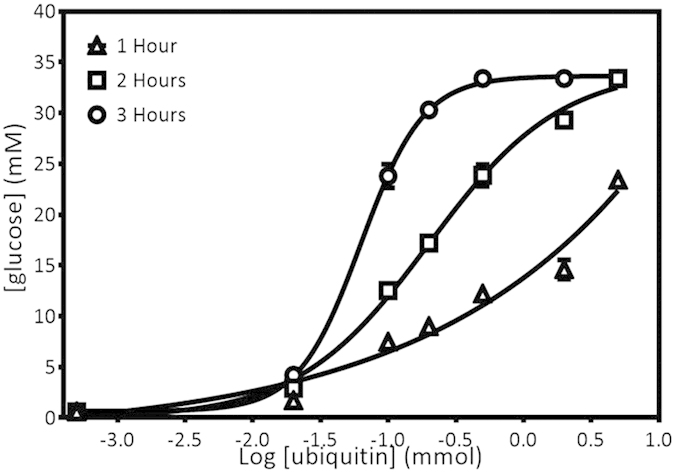
Detection limit of ubiquitin using conjugated Ubi-scFv-LPETG_5_-invB in 1 hour interval. X-axis shows the log titration of ubiquitin detected using Ubi-scFv-LPETG_5_-invB in 1 hr interval. Y-axis shows the reading taken using AccuChek® Performa.

The setup for the application of invB on a PGM for sensing applications differs to that of the eGFP sensing setup. This is due to the fact that several factors such as amount of sucrose template, incubation time and amount of conjugated product required plays an important role in determining the detection limit of the sensor. The amount of sucrose used was fixed at 0.5 M to ensure the amount of sucrose converted to glucose did not exceed the detection limit of the PGM. The considerations for not using higher amounts of sucrose in the reaction was to avoid non-specific conversion and to balance the amount of sucrose present with the amount of invB accessible. A conventional enzyme-linked immunosorbent assay of antibody-conjugated invB (Ubi scFv-LPETG_5_-invB) to the antigen (ubiquitin) was performed to investigate the functionality of the conjugated product after sortase A reaction. The conjugated product at 0.06 nmol was selected as the maximum amount required to generate the read out. The reaction was able to generate a readout at 30 min. However the readout at 1 hr was 17 mmol/L which is at the middle range of the detection limit of the PGM. The amount of glucose converted exceeded the limit of detection of the PGM at 2 hrs making it impossible to determine the actual reading. The reaction was monitored with the presence of controls to eliminate any possible false positive reading (Fig. 9).

The limit of detection of the assay was determined using a range of Ubi at 0 mol, 0.05 pmol, 0.002 nmol, 0.01 nmol, 0.02 nmol, 0.05 nmol, 0.2 nmol and 0.5 nmol (Fig. 9). The setup for the analysis using invB was using 0.06 nmol of the conjugated Ubi scFv-LPETGG_5_-invB and the time limit was set to 3 h as longer incubation periods are not practical for POC applications. A variation in the incubation time was done as the amount of target antigen was reduced to a lower range. As the amount of antigen was reduced, the amount of conjugated antibodies to bind would also decrease. This would mean that the rate of conversion of sucrose to glucose would be reduced all together. Therefore the use of higher amounts of sucrose would not likely benefit the assay as the amount of enzyme would be the limiting factor. An increase in the incubation time would function to compensate for the amount of converted product needed to generate a readout within the detection limit of the PGM. Based on the analysis, the glucose concentration increased as the amount of coated Ubi increased where sucrose conversion became slower as the amount of invB conjugated antibody binding to the antigen was lower reaching the detection limit of the PGM. In this setup, the limit of detection achieved was 0.02 nmol within the set time limit. A balance between the incubation time, amount of sucrose and conjugate is critical for this assay as the detection limit is dependent on these parameters. A prolonged incubation time would undoubtedly improve the sensitivity as this would allow the enzyme a longer time to carry out the conversion of sucrose. An increase in amount of enzyme in the assay would also have similar effects. The increase of sucrose may not provide a major difference as the amount of conjugate is the limiting factor. The assay was designed as a direct assay which would mean the amount of antigen present is directly proportional to the amount of conjugated invB present. This may not be the case if the assay was designed in a competitive setup. Even so, a trade-off between making the assay appropriate for use in a short time frame without having to use too much materials is necessary for a cost-effective assay. We foresee the need to customize the reaction when carrying out similar applications for other samples as time, substrate and enzyme concentration needs will vary to achieve the targeted sensitivity.

### Competitive assay of conjugated Ubi scFv-LPETG_5_-eGFP

Antigen-antibody complex determination between antigen and tagged antibodies could be interfered by the presence of untagged antibodies in a sample. Hence, competitive ELISA of both antibodies was investigated to deterime the ability of the system to carry out a competitive assay.

**Figure 10:**
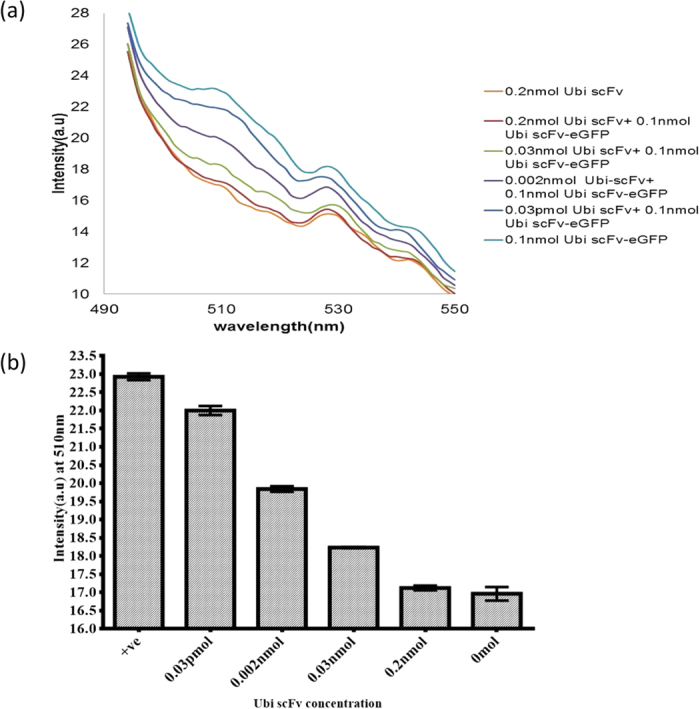
Competitive ELISA of Ubi scFv-eGFP (**a**) Fluorescent reading of competitive assay of the conjugated Ubi scFv-LPETG_5_-eGFP (**b**) Bar chart of fluorescent reading at 510 nm versus amount of Ubi scFv.

In this study, a set of 0 mol, 0.2 nmol, 0.03 nmol, 0.002 nmol and 0.03 pmol of Ubi scFv was used to compete with the conjugated Ubi scFv-LPETG_5_-eGFP to capture Ubi. Figure 10(a) shows the fluorescent reading of the competitive assay utilizing the conjugated Ubi scFv-LPETG_5_-eGFP. For a clearer comparison, a bar chart of fluorescent reading at 510 nm versus amount of Ubi scFv added was plotted (Fig. 10(b)). From the analysis, we found that the fluorescent intensity decreased with increasing inhibition by Ubi scFv in a sample. An almost complete inhibition was recorded in the reaction when maximum amount of Ubi scFv was introduced, generating a weak signal similar to the negative control reaction. The difference between the samples only showed a mean value of 0.161 a.u. The fluorescent signal was still present in the sample containing 0.03 pmol of Ubi scFv giving a difference of 0.93 a.u. mean value to the positive control where 0.1 nmol Ubi scFv-LPETGG_5_-eGFP was allowed to bind to 0.2 nmol Ubi. Therefore, the detection limit of competing antibodies for this assay was determined to be 0.03 pmol.

### Competitive assay of Ubi scFv-LPETG_5_-invB

**Figure 11:**
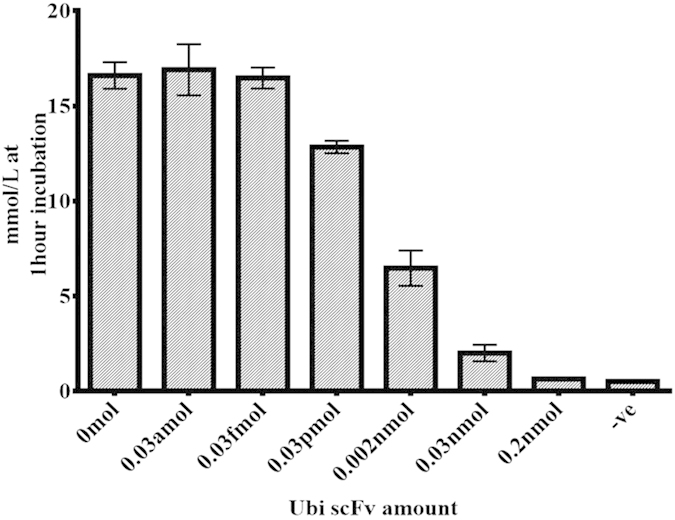
Competitive ELISA of Ubi scFv-invB.

The same approaches of the Ubi scFv-LPETG_5_-eGFP competitive assay was conducted where a set 0 mol, 0.2 nmol, 0.03 nmol, 0.002 nmol and 0.03 pmol of unconjugated Ubi scFv was used as the competitor to the conjugated Ubi scFv-LPETG_5_-invB. Since the amount of the conjugated component was set at 0.06 nmol, the incubation time was limited to only 1 hr. Figure 11 shows the analysis of the competitive assay between Ubi scFv LPETG_5_-invB and unconjugated Ubi scFv. From the analysis, we found that the signal from the PGM decreased as the amount of competing unconjugated Ubi scFv increased. The sample with the addition of 0.2 nmol of Ubi scFv gave the weakest signal with only 0.633 mmol/L which is near the detection limit of the PGM at the lower range. Meanwhile, the glucose conversion in a sample with 0.03 pmol of Ubi scFv was still measureable as the difference to the positive control was 3.767 mmol/L. Further detection of 0.03 fmol and 0.03 amol of Ubi scFv unfortunately were not detectable as the glucose reading did not show any difference in comparison to the positive control sample. Therefore, a range of detection limit for the competitive assay of invB conjugates was determined to be in the 0.03 pmol to 0.03 fmol range. Therefore, a direct fusion of antibodies to invertase by sortase A transpeptidation is able to improve the current technology using PGM as a platform for in-house detection. The sortase A mediated conjugation of recombinant scFv antibodies to invertase would allow for simple swapping of antibodies for the detection of other diseases. The assay is sensitive with low background readings eliminating potential false positive results. The results obtained strengthens the evidence of the ability of conjugated antibodies utilizing the sortase A reaction to maintain its functionality after conjugation for rapid POC assay developments.

## Conclusion

In summary, we have successfully developed a platform for invB-based immunoassay utilizing sortase A mediated conjugation of recombinant antibodies to enzymes. The process was successful at conjugating the two proteins together whilst maintaining its biological functions and structural confirmation. This was seen in both cases where eGFP and invB were used for conjugation. The process allows for simple biology-based conjugation without the need of any chemical modifications to the proteins coupled with a simple two step purification. The assay holds great promise as the conditions can be customized to modify the sensitivity and assay design for a desired diagnostic application using the PGM. Nevertheless, the coupling of sortase based transpeptidation of invB opens a new avenue for the application of PGM as a rapid and cost effective alternative for POC detection of many other diseases in resource limited settings.

## Experimental Methods

### Materials

ElectroMAX^TM^ DH10β^TM^ (Life Technologies), BL21(DE3)(Stratagene) and SHuffle T7®(New England Biolabs) *Escherichia coli* strains were used in this study. pRSET vector was used in all the cloning and expression of recombinant proteins. pQDKG2 aUbi scFv LPETG was a generous gift from Dr. Zoltán Konthur of Max Planck Institute of Colloids and Interfaces. *Staphylococcus aureus* ATCC 25923 was a generous gift from Dr. Venugopal Balakrishnan of Institute for Research in Molecular Medicine.

### Cloning of *eGFP* gene into expression vector and its expression studies

The *eGFP* gene(714 bp) was amplified from pRSET-eGFP and cloned inside pRSET-BH6 at *Nco*I and *Not*I site using primer 5′- CATGCCATGGAGTGAGCAAGGGCGAG GAGCTGTTCACC-3′ and 5′-TTCCTTTTTTGCGGCCGCTGTACAGCTCGTC CATGC-3′. The amplicon was then cloned inside pRSET-BH6 vector using DH10B as the host. The resulting vector was was named pRSET-BH6 eGFP. Expression of eGFP was performed using BL21(DE3) with pRARE-3. Expression condition of biotinylated eGFP was optimized to 25 °C at 160 rpm for 16 hrs with the addition of IPTG (Calbiochem®) at a final concentration of 1 mM and 50 μM D-Biotin(Alfa Aesar®).

### Cloning of *invB* gene into expression vector and its expression studies

The *invB* gene(1201 bp) was synthesized by IDT DNA Technologies following the sequence of the extracellular invertase gene secreted by *Zymomonas mobilis* and amplified using primer 5′- CATCACCATGGGATGGGTGGAGGCTTT-3′ and 5′- AATTAATTAGGGGCCGCATTTGCGACGATCAGG-3′. The amplicon was then cloned inside pRSET-BH6 vector at *Nco*I and *Not*I sites and transformed into DH10B. The resulting vector was named pRSET-BH6 *invB*. Expression of invB was also performed using BL21(DE3) with pRARE-3. Expression condition of biotinylated invB was optimized to 25 °C at 160 rpm for 16 hrs with addition of final IPTG (Calbiochem®) concentration of 1 mM and 50 μM D-Biotin(Alfa Aesar®).

### Design of vector used for Sortase-mediated transpeptidation

For the LPXTG motif, a set of LPETG and LPETGG motif was compared for conjugation efficiency. In this comparative study, pQDKG2 aUbi scFv LPETG and pRSET-B aUbi scFv LPETGG with polyhistidine tag were used. For pRSET-B aUbi scFv LPETGG, the pRSET system was chosen as a cloning and expression vector.

A pentaglycine(G_5_) motif was introduced inside pRSET-BH6 *eGFP* and pRSET-BH6 *invB* by DNA cloning. First, pentaglycine with polyhistidine tag(G_5_H_6X_) nucleotide sequence was converted to double stranded DNA by the filling-in reaction using the Klenow fragment and cloned at the N-terminal of pRSET-BH6 using *NdeI* and *NcoI* restriction site and transformed into DH10B *E.coli* strain. The ssDNA pentaglycine motif used is as followed; 5′-CGCCATATGGGCGGAGGCGGAGGC CACCATCACCATCACCATGGCC-3′. The obtained clone was named as pRSET-G_5_H_6X_ *eGFP* and pRSET-G_5_H_6X_ *invB* were used for transformation into BL21(DE3) for expression studies.

*SrtA* gene was extracted from genomic DNA of *Staphylococcus aureus* and cloned inside pRSET-B vector at *BamHI* and *XhoI* site. Primers used were 5′-CCGTGCTCGAGCTTTATTTGACTTCTGTAGCTAC-3′ and 5′-CGCGGATCCGAAACCACATATCGATAAT-3′. The obtained clone was then transformed inside BL21(DE3) to be expressed.

### Expression and purification of motif-tagged protein and Sortase A

The expression of G_5_-eGFP, G_5_-invB and aUbi scFv-LPETG in BL21(DE3) and aUbi scFv LPETGG in SHuffle T7 was optimized to their optimal condition of 25 °C for 16 hrs at 160 rpm after addition of 1 mM Isopropyl β-D-1-thiogalactopyranoside (IPTG). For sortase A, the optimal condition was obtained at 37 °C for 16 h at 180 rpm using 1 mM IPTG. Cells were harvested by centrifugation at 8000 rpm for 10 min at 4 °C. The cytoplasmic extraction was conducted using 20 μg/mL lysozyme from chicken egg-white followed by sonication process for 3 minutes.

Affinity purification was chosen to purify the proteins where G_5_-eGFP, G_5_-invB, aUbi scFv LPETG, aUbi scFv-LPETGG H6X and sortase A were purified using Nickel-NTA column (GE HealthCare). The expression and purification of target protein were analysed using SDS-PAGE analysis. After that, the purified fractions of the proteins was buffer exchanged to storage buffer of 50 mM Tris and 150 mM NaCl at pH 7.5 except for G_5_-invB which was buffer-exchanged to 50 mM NaOAc and 200 Mm NaCl at pH 5. Each of total protein fractions was concentrated using Vivaspin® 500 Centrifugal Concentrator(GE Healthcare) for 10 min at 15000xg.

### Determining protein concentration

Protein concentration was measured accordingly using Quick Start™ Bradford Protein Assay(Bio-Rad) and a standard curve was plotted. Standard curves with R^2^ = 0.98 to 0.99 only are accepted to be used. Protein concentration was measured based on the obtained equation.

### Optimization of Sortase-mediated conjugation of aUbi scFv-LPETGG with G_5_-eGFP and aUbi scFv-LPETGG with G_5_-invB

The conjugation condition was optimized accordingly starting from motif efficiency and temperature, pH of reaction and CaCl_2_ concentration. All the reactions involved were analysed by SDS-PAGE analysis. Note that all the optimization was conducted using PCR machine (MyCycler, Bio Rad).

#### Motif efficiency and temperature optimization

In a 50 μL reaction, 5 μM of G_5_-eGFP/G_5-_invB, 5 μM aUbi scFv-LPETGG and 5 μM Sortase A were added in buffer condition of 50 Mm Tris, 150 Mm NaCl, 10 mM CaCl_2_ at pH 7.5 to select the best motifs combination and optimal temperature of reaction. The reaction was left for 16 hrs at 4 °C and stopped using 10 mM ethylenediaminetetraacetic acid (EDTA).

#### Optimization of reaction pH

At a selected optimal temperature, a set of reactions containing 5 μM of G_5_-eGFP/ G_5_-eGFP/invB, 5 μM aUbi scFv-LPETGG and 5 μM Sortase A was set up with a variation of buffer condition of 50 mM Tris, 150 mM at pH 6.0, 6.5, 7.0, 7.5, 8.0, 8.5 and 9. The reactions were allowed to proceed at the selected temperature and time.

#### Optimization of CaCl_2_ concentration

At an optimal pH and temperature, reaction was proceeded to find the optimal CaCl_2_ concentration. The same set up of reaction as in (a) and (b) was done but with a variation of CaCl_2_ concentration of 0 mM, 0.5 mM, 1 mM, 2 mM, 5 mM and 10 mM.

#### Optimization of incubation time for reaction

Optimization was continued with incubation from 1, 2, 3, 4, 8 and 16 hrs. The same set up of reaction with the optimal conditions were used and the incubation was stopped with 0.1 Mm EDTA at pH 8.0.

### Purification of conjugated product of Ubi scFv LPETG_5_ eGFP and Ubi scFv LPETG_5_-invB

After an optimized conjugation condition was obtained, two-step affinity purification was conducted to separate pure conjugated product from the remaining unconjugated reactants. To be able to do so, either one of the motif protein must be replaced with other tag. Ubi scFv with LPETGG tag was purified using another system involving the biotin and streptavidin interaction.

Hence, pRSET-B scFv LPETGG was used to clone the avi-tag sequence at the N-terminus using *NdeI* to *NcoI* region. Primers used to amplify the fragment are as followed;5′-GGGAAGCTTCGCCGCCGGTTTCCGGCAGTGCGGCCGCCCGTTTG-3′ and 5′- ACATGCCATGGCCGAGGTGCAGC-3′. The confirmed clone of pRSET-B Avitag aUbi scFv LPETGG was then used for expression using SHuffle T7® *Escherichia coli* at an optimal condition of 25 °C, 160 rpm for 16 hrs using 1 mM IPTG induction with 50 μM D(+)-biotin. The biotinylated aUbi scFv LPETGG was purified using Monomeric Avidin Agarose (Thermo Scientific). The procedure was according to the manufacturer’s instruction without any modification. Eluted biotinylated protein was concentrated using VivaSpin® 20 Centrifugal Concentrator (GE Healthcare) for 16 min at 8000 × g.

A total of 1 mL of 50 μL reactions was prepared and purified accordingly. The purification of conjugated product was conducted using a two-step purification method where both the His-tag and Biotin-tag system was used to eliminate the unreacted reagents thus eluting out the pure conjugated product. First, the reacted product was allowed to pass through the HisTrap FF 1 mL column (GE Healthcare) to trap the Histidine-tagged protein. The purified protein was eluted out using 500 mM Imidazole buffer at pH 7.4. Then, the purified His-tagged product was allowed to pass through a 1 mL column of Monomeric Avidin Agarose (Thermo Scientific) and eluted out using 2 mM D-biotin in 20 mL fraction. Then, the purified fractions were concentrated using Vivaspin® 20 Centrifugal Concentrator (GE Healthcare).

### Western blot analysis of conjugated product

To confirm whether the observed band is the expected conjugated Ubi scFv-LPETG_5_-Egfp/Ubi scFv-LPETG_5_-invB band, a Western blot test was performed. In order to the presence of invB in the conjugated product, anti-His antibody (GE Healthcare) at 1:10,000 was used as the primary antibody and anti-mouse IgG horseradish peroxidase (GE Healthcare) at 1:10,000 was used as the secondary antibody. To test the presence of Ubi scFv-LPETGG, Immmunopure®Protein L, peroxidase conjugated (Pierce Biotechnology) and Pierce High Sensitivity Streptavidin HRP Conjugate at 1:10,000 dilutions was used. Transferring of the protein to the membrane was done using wet transfer system at 100 V for 1 hr. Then, the membrane was blocked for 16 hrs using 5% BSA in PBS + 0.1% Tween 20 with constant shaking. All washing steps were performed using PBS + 0.1% Tween20. After that, primary antibodies were added and allowed to bind for 1 hr. Then secondary antibody was added and incubated for 1 hr before development with Peroxidase Stain DAB kit(Brown Stain) (Nacalai Tesque).

### Limit of detection for Ubi scFv-LPETG_5_-eGFP

A range of 0 mol, 0.05 pmol, 0.001 nmol, 0.002 nmol, 0.005 nmol, 0.01 nmol, 0.02 nmol of Ubi amount were coated inside the wells of Corning Costar® 96-Well EIA/RIA Stripwell™ and blocked with 2% BSA for an hour before addition of 0.1 nmol conjugated Ubi scFv-LPETG_5_-eGFP and allowed to bind for an hour before washing and re-consitute the volume inside the well with 1 × PBS. To measure the fluorescent intensity, Agilent Cary Eclipse Fluorescent spectrophotometer was used. Emission peak of eGFP was determined using excitation taken from 460 nm and emission from 490 nm to 600 nm.

### Kinetics of sucrose conversion by expressed invertase

To test the sucrose conversion activity, a series of 0 nmol, 0.02 fmol, 0.02 pmol, 0.002 nmol, 0.02 nmol and 0.2 nmol of invB was coated on six wells of Corning Costar® 96-Well EIA/RIA Stripwell^TM^ for 1 hr. Then, three wash steps using 1 × PBS + 0.1%Tween 20 was performed before addition of 200 μL of 0.5 M Sucrose. Then, the plate was left incubated at 37 °C for 3 h and the glucose reading was taken at 1 hr intervals using AccuChek® Performa.

### Limit of detection for Ubi scFv-LPETG_5_-invB

0 mol, 0.05 pmol, 0.002 nmol, 0.01 nmol, 0.02 nmol, 0.05 nmol, 0.2 nmol and 0.5 nmol of Ubi were coated on separate wells of Corning Costar® 96-Well EIA/RIA Stripwell™ for 1 hr with 1 × PBS up to 200 μL. The wells was then washed with 1 × PBS with 0.1% Tween 20 for three times and preceded with blocking using 2%BSA for 1 hr. After that, 0.06 nmol of conjugated Ubi scFv LPETG_5_-invB was added together with 2%BSA up to 200 μL for 1 hr. Then the wells were washed for three times. 0.5 M Sucrose in ultrapure water was added into each well and incubated at 37 °C. The glucose reading was measured by using a PGM (AccuChek® Performa, Roche) at 30 min intervals.

### Competitive assay of Ubi scFv-LPETG-eGFP

0.2 nmol of ubiquitin was added into six well of Nunc^TM^ FluoroNunc^TM^/LuminNunc^TM^ 96-Well Plates and incubated for 1 h in shaking condition. After washing the wells with 1XPBS + 0.1%Tween 20 for three times, 2% BSA in 1 × PBS + 0.1% Tween 20 was added as a blocking step for an hour. The wells were washed again before addition of 0 mol, 0.2 nmol, 0.03 nmol, 0.002 nmol, 0.03 pmol of untagged Ubi scFv and incubated before addition of 0.1 nmol of Ubi scFv-LPETG_5_-eGFP into the reaction wells. After incubation for 1 hr, wash step was performed and 200 μL of 1 × PBS was added to the wells before taking the fluorescence reading.

### Competitive assay of Ubi scFv-LPETG_5_-invB

0.2 nmol of Ubi in 1 × PBS was coated in 6 wells of Nunc^TM^ FluoroNunc^TM^/LuminNunc^TM^ 96-Well Plates and incubated for 1 hr in shaking condition before proceeding to wash step using 1 × PBS with 0.1%Tween-20 and finally blocked for 1 hr using 2%BSA in 1 × PBS + 0.1%Tween 20. Untagged Ubi scFv was then added at a concentration of 0 mol, 0.2 nmol, 0.03 nmol, 0.002 nmol and 0.03 pmol after performing the wash step. Incubation was done for 1 hr before addition of 0.06 nmol of Ubi scFv-LPETG_5_-invB into each wells and left to incubate for 1 hr. A wash step was performed before adding 0.5 M Sucrose and incubated for 1 hr before the readings on the PGM were taken.

## Additional Information

**How to cite this article**: Ismail, N. F. and Lim, T. S. Site-specific scFv labelling with invertase via Sortase A mechanism as a platform for antibody-antigen detection using the personal glucose meter. *Sci. Rep.* **6**, 19338; doi: 10.1038/srep19338 (2016).

## Supplementary Material

Supplementary Information

## Figures and Tables

**Figure 1:**
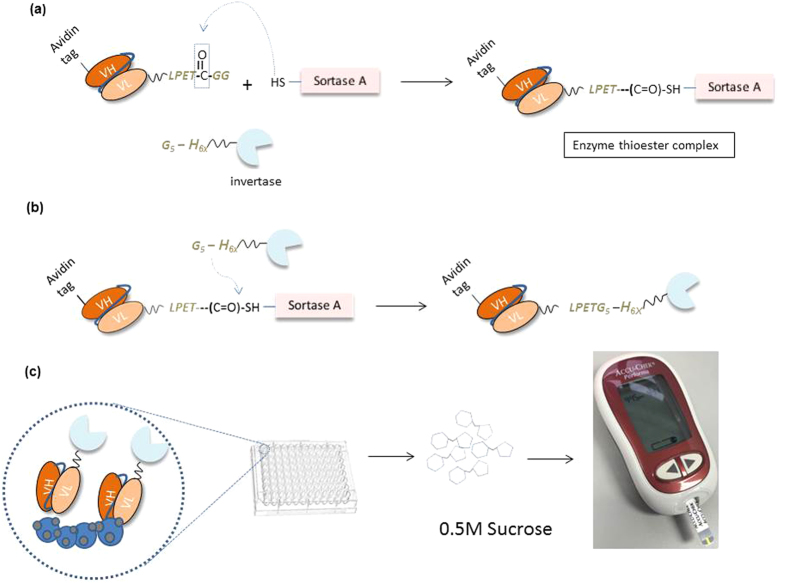
Conjugation of Ubi scFv-LPETGG with G_5_-invB processes and invB assay. (**a**) Mechanism of Sortase A recognizes the LPXTG motif on Ubi scFv-LPETGG and forming the intermediate complex (**b**) Mechanism of G_5_-invB form an amide bond with Ubi scFv-LPETGG and releasing the free Sortase A. (**c**) invB assay using conjugated product of Ubi **scFv**-LPETG_5_-invB.

## References

[b1] Van Weemen B. K. & Schuurs A. H. W. M. Immunoassay using antigen—enzyme conjugates. FEBS Lett. 15, 232–236 (1971). 11945853 10.1016/0014-5793(71)80319-8

[b2] Wang J. Electrochemical biosensors: Towards point-of-care cancer diagnostics. Biosens. Bioelectron. 21, 1887–1892 (2006). 16330202 10.1016/j.bios.2005.10.027

[b3] Backmann N. *et al.* A label-free immunosensor array using single-chain antibody fragments. Proc Nat Acad Sci USA 102, 14587–14592 (2005). 16192357 10.1073/pnas.0504917102 PMC1253559

[b4] Grandke J., Oberleitner L., Resch-Genger U., Garbe L. A. & Schneider R. J. Quality assurance in immunoassay performance—comparison of different enzyme immunoassays for the determination of caffeine in consumer products. Anal. Bioanal. Chem 405, 1601–1611 (2013). 23224576 10.1007/s00216-012-6596-0

[b5] Schaap A., Akhavan H. & Romano L. Chemiluminescent substrates for alkaline phosphatase: application to ultrasensitive enzyme-linked immunoassays and DNA probes. Clin. Chem. 35, 1863–1864 (1989). 2505948

[b6] De Noya B. A. *et al.* Evaluation of alkaline phosphatase immunoassay and comparison with other diagnostic methods in areas of low transmission of schistosomiasis. Acta tropica 66, 69–78 (1997). 9227799 10.1016/s0001-706x(97)00032-6

[b7] Dai L. *et al.* Development of a potential high-throughput workflow to characterize sites of bioconjugation by immuno-affinity capture coupled to MALDI-TOF mass spectrometry. Bioconjug Chem 24, 53–62 (2013). 23186023 10.1021/bc300413c

[b8] Tresca J. P., Ricoux R., Pontet M. & Engler R. Comparative activity of peroxidase-antibody conjugates with periodate and glutaraldehyde coupling according to an enzyme immunoassay. Ann Biol Clin 53, 227–231 (1995). 7574110

[b9] Carmenate T. *et al.* Effect of conjugation methodology on the immunogenicity and protective efficacy of meningococcal group C polysaccharide–P64 k protein conjugates. FEMS Immunol Med Microbiol 40, 193–199 (2004). 15039094 10.1016/S0928-8244(03)00346-8

[b10] Kolb H. C., Finn M. G. & Sharpless K. B. Click Chemistry: Diverse Chemical Function from a Few Good Reactions. Angew. Chem. Int. Ed. 40, 2004–2021 (2001). 10.1002/1521-3773(20010601)40:11<2004::AID-ANIE2004>3.0.CO;2-5 11433435

[b11] Kolb H. C. & Sharpless K. B. The growing impact of click chemistry on drug discovery. Drug Discovery Today 8, 1128–1137 (2003). 14678739 10.1016/s1359-6446(03)02933-7

[b12] Moses J. E. & Moorhouse A. D. The growing applications of click chemistry. Chem. Soc. Rev. 36, 1249–1262 (2007). 17619685 10.1039/b613014n

[b13] Bentley M. L., Gaweska H., Kielec J. M. & McCafferty D. G. Engineering the substrate specificity of *Staphylococcus aureus* Sortase A. The Beta6/beta7 loop from SrtB confers NPQTN recognition to SrtA. J. Biol. Chem. 282, 6571–6581 (2007). 17200112 10.1074/jbc.M610519200

[b14] Bentley M. L., Lamb E. C. & McCafferty D. G. Mutagenesis studies of substrate recognition and catalysis in the sortase A transpeptidase from *Staphylococcus aureus*. J. Biol. Chem. 283, 14762–14771 (2008). 18375951 10.1074/jbc.M800974200 PMC2386945

[b15] Ta H. T. *et al.* Enzymatic Single-Chain Antibody Tagging: A Universal Approach to Targeted Molecular Imaging and Cell Homing in Cardiovascular Disease. Circulation Research 109, 365–373 (2011). 21700932 10.1161/CIRCRESAHA.111.249375

[b16] Mao H., Hart S. A., Schink A. & Pollok B. A. Sortase-Mediated Protein Ligation: A New Method for Protein Engineering. J. Am. Chem. Soc. 126, 2670–2671 (2004). 14995162 10.1021/ja039915e

[b17] Parthasarathy R., Subramanian S. & Boder E. T. Sortase A as a Novel Molecular “Stapler” for Sequence-Specific Protein Conjugation. Bioconjugate Chem. 18, 469–476 (2007). 10.1021/bc060339w 17302384

[b18] Tanaka T., Yamamoto T., Tsukiji S. & Nagamune T. Site-specific protein modification on living cells catalyzed by Sortase. Chembiochem 9, 802–807 (2008). 18297670 10.1002/cbic.200700614

[b19] Popp M. W., Antos J. M., Grotenbreg G. M., Spooner E. & Ploegh H. L. Sortagging: a versatile method for protein labeling. Nat Chem Biol 3, 707–708 (2007). 17891153 10.1038/nchembio.2007.31

[b20] Xiang Y. & Lu Y. Using personal glucose meters and functional DNA sensors to quantify a variety of analytical targets. Nature Chem 3, 697–703 (2011). 21860458 10.1038/nchem.1092 PMC3299819

[b21] Xiang Y. & Lu Y. Portable and Quantitative Detection of Protein Biomarkers and Small Molecular Toxins Using Antibodies and Ubiquitous Personal Glucose Meters. Anal. Chem. 84, 4174–4178 (2012). 22455548 10.1021/ac300517n PMC3341531

[b22] Xiang Y. & Lu Y. Using Commercially Available Personal Glucose Meters for Portable Quantification of DNA. Anal. Chem. 84, 1975–1980 (2012). 22235863 10.1021/ac203014s PMC3302979

[b23] Xiang Y. & Lu Y. An invasive DNA approach toward a general method for portable quantification of metal ions using a personal glucose meter. Chem. Commun. 49, 585–587 (2013). 10.1039/c2cc37156a PMC3765066 23208450

[b24] Yan L. *et al.* Target-responsive “sweet” hydrogel with glucometer readout for portable and quantitative detection of non-glucose targets. J. Am. Chem. Soc. 135, 3748–3751 (2013). 23339662 10.1021/ja3114714

[b25] Vásquez-Bahena J. M. *et al.* Expression and improved production of the soluble extracellular invertase from *Zymomonas mobilis* in *Escherichia coli*. Enzyme Microb. Technol 40, 61–66 (2006).

[b26] O’Mullan P. J., Chase T. Jr & Eveleigh D. E. Purification and some properties of extracellular invertase B from *Zymomonas mobilis*. Appl. Microbiol. Biotechnol. 38, 341–346 (1992).

[b27] Popp M. W., Antos J. M. & Ploegh H. L. Site-specific protein labeling via sortase-mediated transpeptidation. Curr Protoc Protein Sci Chapter 15 (2009). 10.1002/0471140864.ps1503s56 PMC5551486 19365788

[b28] Theile C. S. *et al.* Site-specific N-terminal labeling of proteins using sortase-mediated reactions. Nat. Protoc. 8, 1800–1807 (2013). 23989674 10.1038/nprot.2013.102 PMC3941705

[b29] Heck T., Pham P.-H., Yerlikaya A., Thöny-Meyer L. & Richter M. Sortase A catalyzed reaction pathways: a comparative study with six SrtA variants. Catal Sci Technol 4, 2946–2956 (2014).

[b30] Mohlmann S., Mahlert C., Greven S., Scholz P. & Harrenga A. *In vitro* sortagging of an antibody fab fragment: overcoming unproductive reactions of sortase with water and lysine side chains. Chembiochem 12, 1774–1780 (2011). 21656631 10.1002/cbic.201100002

[b31] Huang X. *et al.* Kinetic Mechanism of *Staphylococcus aureus* Sortase SrtA. Biochemistry 42, 11307–11315 (2003). 14503881 10.1021/bi034391g

[b32] Bottomley S. P. *et al.* Cloning, expression and purification of Ppl-1, a kappa-chain binding protein, based upon protein L from *Peptostreptococcus magnus*. Bioseparation 5, 359–367 (1995). 8767928

[b33] Murphy J. P. *et al.* Amplified expression and large-scale purification of protein L′. Bioseparation 6, 107–113 (1996). 8818265

[b34] Guesdon J. L., Ternynck T. & Avrameas S. The use of avidin-biotin interaction in immunoenzymatic techniques. J. Histochem. Cytochem. 27, 1131–1139 (1979). 90074 10.1177/27.8.90074

[b35] Izrailev S., Stepaniants S., Balsera M., Oono Y. & Schulten K. Molecular dynamics study of unbinding of the avidin-biotin complex. Biophys. J. 72, 1568 (1997). 9083662 10.1016/S0006-3495(97)78804-0 PMC1184352

[b36] Levary D. A., Parthasarathy R., Boder E. T. & Ackerman M. E. Protein-protein fusion catalyzed by Sortase A. PloS one 6 (2011). 10.1371/journal.pone.0018342 PMC3071835 21494692

[b37] Popp M. W. & Ploegh H. L. Making and breaking peptide bonds: protein engineering using sortase. Angew. Chem. Int. Ed. Engl. 50, 5024–5032 (2011). 21538739 10.1002/anie.201008267

[b38] Kay B., Thai S. & Volgina V. In High Throughput Protein Expression and Purification Vol. 498, Methods Mol. Biol. (ed Sharon A. Doyle) Ch. 13, 185–198 (Humana Press, 2009). 18988027 10.1007/978-1-59745-196-3_13 PMC3223083

