# Supplementary material for: Site-specific scFv labelling with invertase via Sortase A mechanism as a platform for antibody-antigen detection using the personal glucose meter
Source: Sci Rep. 2016 Jan 19;6:19338. doi: 10.1038/srep19338 (PMC4726117; doi:10.1038/srep19338)
Supplement: Supplementary Information [file srep19338-s1.doc]

**Supporting information**

**Site-specific scFv labelling with invertase via Sortase A mechanism as a platform for antibody-antigen detection using the personal glucose meter**

**Nur Faezee Ismail^1^ and Theam Soon Lim^1^,***

^1^ Institute for Research in Molecular Medicine, Universiti Sains Malaysia, 11800 Penang, Malaysia

*[theamsoon@usm.my](mailto:theamsoon@usm.my)

**1. Sortase A-mediated conjugation of Ubi scFv-LPETGG to G_5_-eGFP.** eGFP (26.4kDa) was used as a control for Sortase A transpeptidation to ensure the system established is properly working. Therefore, eGFP which act as a reporter protein was used to conjugate to Ubi scFv (29.97kDa) before proceed to conjugation of invB to Ubi scFv. In this study, eGFP was chosen to be tagged with oligoglycine motif; meanwhile, Ubi scFv was tagged with LPXTG motif. The optimization of conjugation condition involved was referred to the previous study^1-3^.

*1.1 Optimization of motif efficiency and temperature of conjugation*

As temperature and efficiency of the motif are the important parameters, variation in the temperature of reaction was done from the lowest to highest temperature at 4˚C to 50˚C for 3 hours (hrs) incubation time using the conjugation of tri-glycine motif eGFP and penta-glycine motif protein. Figure 1 shows the comparison of conjugation yield aided by Sortase A at temperature ranging from 4˚C to 50˚C. From the analysis, only the conjugation of G_5_-eGFP and Ubi scFv-LPETGG consistently gave a thick band at the estimated conjugated product size of 56.4kDa at every tested temperature. Since all of the tested temperature gave almost the same intensity of bands, reaction of Ubi scFv-LPETGG and G5-eGFP at 37˚C was selected as the efficient motifs and optimal temperature.


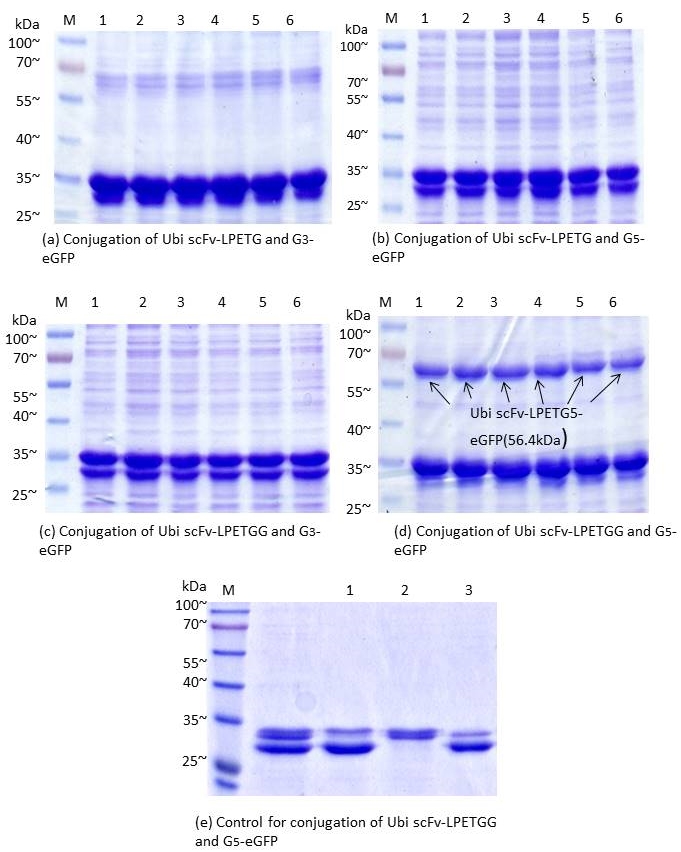


**Figure 1: Optimization of motif efficiency and temperature of conjugation.(a)-(d)** M: BluElf Prestained Protein Ladder, 1: reaction at 4˚C,2, reaction at 16˚C, 3: reaction at 25˚C, 4: reaction at 37˚C, 5: reaction at 45˚C, 6: reaction at 50˚C. **(e)** M: BluElf Prestained Protein Ladder, 1: reaction without Ubi scFv-LPETGG, 2: reaction without G_5_-eGFP, 3: reaction without SrtA

*1.2 pH optimization of conjugation*

pH optimization was done since some of the protein depends on pH of the reaction to initiate the conjugation process. A series of pH of buffer from 6.0 to 9.0 were tested. For the conjugation of Ubi scFv and eGFP, the efficiency of the conjugation was consistent in all of the conjugation reaction. pH7.5 was selected as the conjugation of Ubi scFv and eGFP was not affected by pH of reaction. Figure 2(a) shows the pH optimization of conjugation of Ubi scFv-LPETGG and G_5_-eGFP.

*1.3 Optimization of CaCl_2_ concentration*

CaCl_2_ is an important component in the Sortase A mechanism as it helps in the stabilization of the conjugation reaction. Therefore, a range of CaCl_2_ concentration of 0, 1, 2, 5 and 10mM of CaCl_2_ were tested to optimize the conjugation yield. Figure 2(b) shows the CaCl_2_ concentration used in the conjugation of Ubi scFv-LPETG_5_-eGFP. In this study, 5mM of CaCl_2_ was chosen as the optimal concentration of CaCl_2_ of the conjugation of Ubi scFv-LPETGG and G_5_-Egfp.

*1.4 Optimization of incubation time*

After a suitable conjugation condition established, we seek the opportunities to increase the conjugation yield by varying the incubation time of the reaction. Hence, a set of reactions was set up and allowed to conjugate at different incubation time at two different temperature, 4˚C and 37˚C using two concentration of enzyme, 1:1 ratio of enzyme to reactant and 5:1 of enzyme to reactant. Figure 2(c) shows the Ubi-scFv-LPETGG and G_5_-eGFP conjugation performed at different incubation time using the optimized condition. Based on the results, shorter incubation time and higher temperature almost the same band intensity to the prolong incubation time and low temperature of reaction. Hence, 37˚C and 3 hrs incubation was selected as the optimal incubation time.


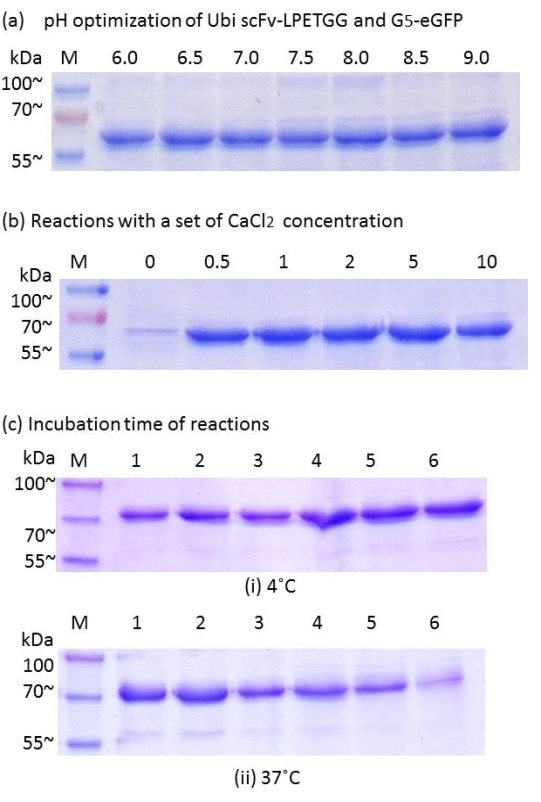


**Figure 2: Optimization of Ubi scFv-LPETGG and G_5_-eGFP conjugation. (a)** pH optimization of Ubi scFv-LPETGG and G_5_-eGFP conjugation **(b)** CaCl_2_ optimization of Ubi scFv-LPETGG and G_5_-eGFP conjugation, **(c)** Incubation time of reactions.

**2. Sortase A-mediated conjugation of Ubi scFv-LPETGG to G_5_invB**. We describe here the *in vitro* conjugation of Ubi scFv (29.97kDa) and invB (45.8kDa) via sortase-mediated transpeptidation. Ubi scFv was tagged with LPXTG motif either LPETG or LPETGG motif at C-terminus of the protein. Meanwhile, pentaglycine(G_5_) motif was introduced at the N-terminus of i*nvB*.

*2.1 Optimization of motif efficiency and temperature of conjugation*

To find the optimal temperature with an efficient motif, we systematically vary the temperatures for conjugation from the lowest to highest temperature at 4ºC to 50ºC for 3 hrs using two different LPXTG motif protein. Figure 3 shows the comparison of conjugation yield aided by Sortase A at various temperature using two types of LPXTG motif protein. The estimated band size of ~75.8kDa was present at all lanes of 3(b), however, a very little amount of expected band size was observed in 3(a) which is the reaction using scFv with LPETG tag. The yield of conjugated Ubi scFv and invB is intensely reduced in 3(a) despite the concentration of both protein are kept constant between both set of reactions. This indicates conjugation of Ubi scFv to invB via sortase A is directly affected by the efficiency of motif-motif interaction which the addition of extra glycine of the LPXTG motif protein increased the chance of conjugation to happens as suggested by Theile, et al. ^4^.


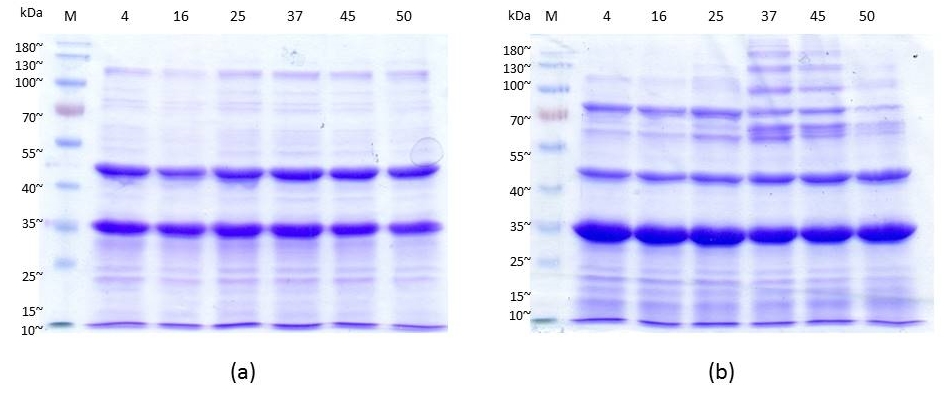


Ubi scFv-LPETG_5_-invB(75.8kDa)

**Figure 3:** **SDS-PAGE analysis of comparison of conjugation yield aided by Sortase A at various temperature using two types of LPXTG motif protein. (a)**Ubi scFv-LPETG and invB conjugation at various temperatures. **(b)** Ubi scFv-LPETGG and invB conjugation at various temperatures. M: Marker (Thermo Scientific Prestained Protein Ladder). Lane 1 to 6 shows the reaction performed at 4ºC to 50ºC.

As Ubi scFv-invB yield is improved using Ubi scFv-LPETGG motif, we move on to study the temperature effects on conjugation yield. Based on Figure 3(b), the yield is slightly better and more specific at low temperature ranges from 4ºC to 25ºC where the expected band is more intense and less amount of contaminant bands is observed. The conjugation was less specific with higher temperatures lower the yields of conjugated product. The reaction lane representing reaction at 37ºC to 50ºC shows a decrement in conjugation yield and also specificity. More unidentified bands were observed in those temperatures. Hence, a suitable temperature for reaction must be selected from the range of 4ºC to 25ºC. 25ºC was selected as the optimal temperature using scFv with LPETGG motif due to the yield is more than the other temperatures with acceptable specificity.

*2.2 pH optimization of conjugation*

Since invB is a pH dependent protein ^5-7^, optimization of pH in buffer condition is essential for the production of functional conjugated products. In our experiences, invB tend to precipitates in neutral to basic solution causing destabilization to its structure thus affecting the conjugation reaction. This observation might be attributed to the acidic properties of invB ^8^. Therefore, a variation of reaction pH was used to test the suitability of buffer conditions for the hydrolysis of sucrose to glucose afterwards and also improvement in conjugation yield . Invertases generally exist as an acidic protein in nature^8^. Hence, a set of buffer ranging from pH 6 to 9 was prepared. Figure 4(a) shows the conjugation of Ubi scFv-LPETGG and G_5_-invB performed in different buffer conditions. From pH 6 to 9, the conjugation yield is lessening as the pH of the solution increases. Thus, we can conclude conjugation reaction of Ubi scFv and invB is strongly affected by pH. Based on the findings, the reaction is more efficient in a slightly acidic condition. Therefore, pH 6.0 was chosen as the optimal pH condition for conjugating Ubi scFv to invB.

*2.3 Optimization of CaCl_2_ concentration*

Sortase A isolated from *Staphylococcus aureus* is a calcium dependent type of sortase. The calcium ion act as the kosmotropic reagent for sortase activity where it helps in stabilizing the substrate binding to the active site of sortase A, cysteine-184. However, the concentration of CaCl_2_ must be added in a right amount so that the reaction is not too slow but not too much as it will facilitate the dissociation of conjugated product. Hence, a range of CaCl_2_ concentrations were tested to compare the conjugation yield. Figure 4 (b) shows the optimization of CaCl_2_ concentration in two different pH, (i) pH7.5 and (ii) pH6.0. In both condition, addition of 0.5mM to 10mM CaCl_2_ gave a relatively same amount of conjugated product, with a slight difference at 5mM CaCl_2_. Therefore, 5mM CaCl_2_ was chosen as the optimal concentration for the conjugation of scFv and invB.

*2.4 Optimization of incubation time*

After a suitable conjugation condition established, we seek the opportunities to increase conjugation yield by varying the incubation time of the reaction. Hence, a set of reactions was set up and allowed to conjugate at different incubation time. Figure 4(c) shows the Ubi-scFv-LPETGG and G_5_-invB conjugation performed at different incubation time using the optimized condition. From 1 hr to 4 hrs, the conjugation yield constantly gave relatively same intensity of conjugated Ubi scFv-invB band. At 8 hrs and 16 hrs incubation time, the intensity of conjugated Ubi scFv-invB band decreased. These results support the theory of reversible sortase A aided-conjugation where at prolong incubation time, when the maximum amount of conjugated products are achieved, the amount of conjugated product depleted as a result of the reverse reaction of Sortase A ^9^.


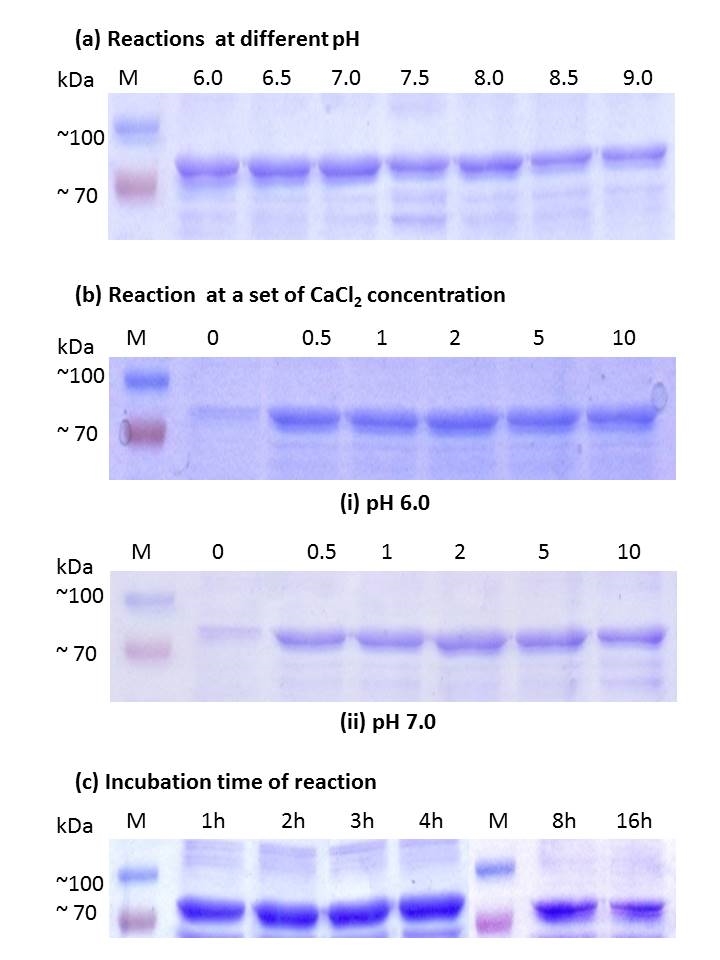


**Figure 4: Optimization of Ubi-scFv-LPETGG and G_5_-invB conjugation. (a)** Optimization of pH of reaction, **(b)** Optimization of CaCl_2_ concentration at two different pH, (i) pH 6.0 (ii) pH 7.5 , **(c)** Optimization of incubation time of reaction

Figure 5(a) shows the conjugation of Ubi scFv and invB via Sortase-mediated conjugation at the optimal condition of 25ºC, pH6.0 with 5mM CaCl_2_ and 3 hrs incubation time. Lane 1 represents the reaction lane where unconjugated Ubi scFv-LPETGG size calculated was ~29.97kDa and the size of unconjugated G_5_-invB was ~45.8kDa. The expected size of Sortase A is ~23.75kDa. However, in the SDS-PAGE analysis, we observed the Sortase A band to be slightly higher than expected size. For conjugated product, the expected size is at 75.8kDa. As we can see, with respect to all the controls, only reaction at lane 1 produces the expected conjugates band. Therefore with acceptable amount of conjugated Ubi scFv-invB, the optimized condition is so far the best condition for Ubi scFv-invB conjugation via Sortase A enzymatic activity.

**References**

1 Popp, M. W., Antos, J. M., Grotenbreg, G. M., Spooner, E. & Ploegh, H. L. Sortagging: a versatile method for protein labeling. *Nat Chem Biol* **3**, 707-708 (2007).

2 Popp, M. W., Antos, J. M. & Ploegh, H. L. Site-specific protein labeling via sortase-mediated transpeptidation. *Current Protocols in Protein Science* **56**,15.3:15.3.1–15.3.9 (2009).

3 Ta, H. T. *et al.* Enzymatic single-chain antibody tagging: a universal approach to targeted molecular imaging and cell homing in cardiovascular disease. *Circ Res* **109**, 365-373 (2011).

4 Theile, C. S. *et al.* Site-specific N-terminal labeling of proteins using sortase-mediated reactions. *Nat Protoc* **8**, 1800-1807 (2013).

5 Sturm, A. Invertases. Primary Structures, Functions, and Roles in Plant Development and Sucrose Partitioning. *Plant Physiology* **121**, 1-8 (1999).

6 Xiang, L. *et al.* Exploring the neutral invertase–oxidative stress defence connection in *Arabidopsis thaliana*. *J. Exp. Bot.* **62**, 3849-3862 (2011).

7 Du, L. *et al.* Characterization of an Invertase with pH Tolerance and Truncation of Its N-Terminal to Shift Optimum Activity toward Neutral pH. *PloS one* **8,** e62306 (2013).

8 O'Mullan, P. J., Chase Jr, T. & Eveleigh, D. E. Purification and some properties of extracellular invertase B from *Zymomonas mobilis*. *Appl. Microbiol. Biotechnol.* **38**, 341-346 (1992).

9 Huang, X. *et al.* Kinetic Mechanism of *Staphylococcus aureus* Sortase SrtA. *Biochemistry* **42**, 11307-11315 (2003).
